# The Water Polo Intermittent Shuttle Test in Women's Water Polo Players

**DOI:** 10.1002/ejsc.70074

**Published:** 2025-10-30

**Authors:** Mico H. Olivier, Adam D. Gorman, Mark J. Connick, Patrick M. Holmberg, Jordan Desbrow, Vincent G. Kelly

**Affiliations:** ^1^ Faculty of Health School of Exercise and Nutrition Sciences Queensland University of Technology Brisbane Australia; ^2^ Centre for Sport Performance Innovation and Knowledge Excellence (SPIKE) Queensland Academy of Sport Brisbane Australia; ^3^ Performance Support Department Queensland Academy of Sport Brisbane Australia

**Keywords:** aquatic‐sports, fitness outcomes, performance goals, physiological testing, reliability

## Abstract

This study aimed to (a) establish the test‐retest reliability of the water polo intermittent shuttle test (WIST) in elite female water polo players, (b) investigate the validity of the WIST to determine positional differences in WIST scores within this population and (c) distinguish between competitive female playing standards. Part one involved 14 elite female water polo players (24.2 ± 3.2 years, experience > 5 years) completing the WIST on two separate occasions, separated by 48 h. In part two, 18 elite (24.4 ± 3.5 years), 7 highly trained (21.6 ± 3.2 years) and 34 trained (13–17 years) female water polo players completed the WIST. The coefficient of variation (CV), intraclass correlation coefficient (ICC), smallest worthwhile change (SWC) and minimal detectable change 90% CI (MDC_90_) were calculated. The WIST demonstrated acceptable reliability (ICC = 0.93, CV = 6.7%) and usefulness to detect performance changes with SWC_0.2_ (22.3 m) > TE 90% CI [(17.5 m (11.6, 23.3))], quantifying practically meaningful changes in performance (MDC_90_ = 54.8 m or ∼ 4 shuttles). No significant positional differences were evident. Very large, positive, statistically significant differences were found between highly trained and trained (U14 to U17) players (*p <* 0.01, *g* = 3.1) and between elite and trained (U14 to U17) players (*p <* 0.01, *g* = 2.4). The WIST is a reliable and useful high‐intensity intermittent performance test suitable for elite female water polo players. Differences across standards of competition confirmed the sensitivity and validity of the WIST. Practitioners can quantify practically meaningful changes in WIST performance using MDC_90_.

## Highlights

- The water polo intermittent shuttle test (WIST) is a reliable physiological performance test to be used in elite women's water polo players.
- Practitioners using the WIST as part of a physiological testing battery can detect and quantify practically meaningful changes in performance outcomes.
- Distinct differences in WIST performance across standards of competition confirmed the discriminatory ability of the test and allowed tailored interventions to be designed in accordance with the demands of each competitive playing level.

## 1 Introduction

Well‐developed aerobic power and cardiorespiratory endurance are required to meet the high‐intensity activity demands of water polo competition (Botonis et al. 2019). Elite male players generally swim ∼54–60 m per minute and total distances of ∼1600–1750 m across 4 × 8 min quarters, with 19%–25% of this performed at high‐intensity (1.4–1.8 m.s^−1^) and very high‐intensity (> 1.8 m.s^−1^) swimming (Botonis et al. 2019; Melchiorri, Castagna, et al. 2010). Additional high‐intensity activities during games involve active offensive (e.g., driving, faking, shooting, passing, drawing fouls) and defensive activities (e.g., blocking, jumping, defending drives, committing fouls) and wrestling bouts. These activities vary between 2.6 and 15.4 s and often are interspersed with lower‐intensity activities (e.g., easy crawl, slow speed swimming, treading water) lasting 20 s, with work:rest ratios ranging from ∼1:1.6–7 (Botonis et al. 2019; Platanou 2004; Platanou and Geladas 2006; Smith 1998). The intermittent, high‐intensity activities demonstrated during elite‐level competition have been shown to elicit substantial physiological responses, with men's water polo players' heart rates typically > 160 b. min^−1^, corresponding to 85% of their peak heart rate (Botonis et al. 2019; Galy et al. 2014; Pinnington et al. 1988; Platanou and Geladas 2006). Given the importance of these high‐intensity activities, performance tests that reflect the intermittent nature of match play and mimic the cardiometabolic demands of water polo can provide insight into players' physiological capacities and inform subsequent training prescriptions.

Direct assessment of players' physiological capacities is generally difficult in applied settings, where there is typically minimal equipment, limited qualified personnel, budgetary restrictions and large groups (Ramsbottom et al. 1988). Recently, Chirico et al. (Chirico et al. 2022) conducted a systematic review to illustrate the scope of physiological swimming tests employed by performance staff in water polo. For instance, the multistage shuttle swim test (MSST), requiring individuals to swim a 10‐m distance with progressively faster speeds every minute, was developed to specifically assess the aerobic fitness levels of trained male and female water polo players (Rechichi et al. 2000). Despite performance outcomes correlating well with maximal oxygen uptake as determined during an incremental tethered swim test to exhaustion (Rechichi et al. 2000), the assessment did not account for the intermittent activity patterns evident during match play (Mujika et al. 2006). Thus, the validity of the MSST to assess players' physiological capacity to meet the intermittent competitive demands is somewhat limited (Mujika et al. 2006). Understanding these limitations, Mujika et al. (Mujika et al. 2006) developed the water polo intermittent shuttle test (WIST), based on the Yo‐Yo Intermittent Recovery Test (Level 1), given its ability to evaluate repeated intermittent activities or intense efforts (Bangsbo et al. 2008), involving repeated 2 × 7.5 m swims out and back at progressively increasing speeds with brief periods (10 s) of active recovery. The WIST was demonstrated to be a reliable and valid test for trained male water polo players, as some physiological indices (i.e., heart rate [HR] and lactate) were comparable to those observed during competitive matches, and the results were strongly correlated with coaches’ subjective match fitness rankings (Mujika et al. 2006). Furthermore, the WIST demonstrated a high sensitivity for detecting performance differences among playing positions, between field players and goalkeepers, and for competitive standards and monitoring seasonal fitness changes (Mujika et al. 2006). However, all players, males and females, were pooled together across all competitive levels during the analysis; thus, it is unknown whether the differences exist within elite female water polo competitors. Similarly, the measurement error associated with the WIST, and therefore its ability to assess practically meaningful changes in high‐intensity intermittent performance, has yet to be determined.

The modification of match rules and the development of training processes over the past 20 years have made the game more demanding (Li and Graham 2021; Melchiorri, Castagna, et al. 2010; Paixão et al. 2023). As such, there is increased importance placed on the capacity to perform repeated high‐intensity activities with short recovery periods (Botonis et al. 2019; D’Auria and Gabbett 2008; Melchiorri, Castagna, et al. 2010). Additionally, the growth of women’s water polo since it was initially included in the Olympic programme in 2000, combined with further rule changes in the last decade to make the sport more dynamic, has intensified competitive demands, as evidenced by more frequent changes in movement patterns [from every 7.4 (D’Auria and Gabbett 2008) to 6.2 (Tan et al. 2009a) seconds] and greater shots attempted at goal during counterattack situations (Argudo‐Iturriaga et al. 2020). The increasing physiological demands of women’s water polo match play require the WIST to be re‐examined to determine whether it remains a useful tool to assess match fitness in elite female players. Only Mujika et al. (Mujika et al. 2006) has investigated measurement ‘noise’ (typical error [TE]) during the WIST, with no study having examined the smallest worthwhile change (SWC) to determine the practicability of the test to monitor player progression (Buchheit et al. 2010). Furthermore, no investigations have assessed the minimal detectable change signal (MDC) in the WIST, making it difficult to determine whether clear changes in players’ physiological capacities have occurred after periods of training, nor have any studies established the reliability of the assessment (and its discriminative ability) in female competitors. Considering the few studies investigating the match demands of women’s water polo and limited available data showing applicable fitness qualities of female players (Botonis et al. 2019; D’Auria and Gabbett 2008; Platanou 2009; Platanou and Varamenti 2011; Radovanovic et al. 2007; Tan et al. 2009a; Varamenti and Platanou 2008), further research examining these parameters in elite female competitors is warranted (Staurowsky et al. 2020).

Water polo positions involve distinct movement patterns and high‐intensity demands (D’Auria and Gabbett 2008; Tan et al. 2009a). For example, drivers perform more frequent and longer swimming efforts, whereas centre positions (centre forward and centre back) are more involved in high‐intensity wrestling activities (Tan et al. 2009a). Accordingly, cardiorespiratory demands have been shown to vary by both position and competition level (Botonis et al. 2016). A higher aerobic capacity offers performance benefits, including the ability to sustain higher intensities during matches while attenuating decreases in work rate and technical performance, such as ball shooting accuracy and velocity (Botonis et al. 2019; Galy et al. 2014). Physiological profiles have been used to identify characteristics that may help individuals meet the demands of water polo competition (Platanou and Varamenti 2011; Tan et al. 2009b; Varamenti and Platanou 2008). Accordingly, Tan et al. (Tan et al. 2009b) previously identified hierarchical differences in high‐intensity intermittent performance and physiological measures across competitive levels using the MSST and WIST. However, differences in these measures have yet to be examined among playing positions in female competitors. Moreover, further research is needed to determine whether WIST performance outcomes differ across competitive levels in women’s water polo.

This study aimed to (a) establish the test‐retest reliability, standard error of measurement and minimum detectable change of the WIST in elite female water polo players, (b) determine positional differences in WIST scores within this population and (c) investigate the construct validity of the WIST to distinguish between competitive female playing standards. It was hypothesised that the WIST would be a reliable tool to assess match fitness in elite female water polo players and that WIST scores would differ among positions and competitive levels, with testing outcomes reflecting hierarchical playing standards.

## 2 Methods

### 2.1 Experimental Approach to the Problem

This study was divided into two parts, with part one establishing the test‐retest reliability of the WIST in elite female water polo players while also investigating positional differences in scores and part two investigating differences in WIST scores across different levels of competitive female playing standards.

### 2.2 Part 1—Reliability

#### 2.2.1 Subjects

Sample size was calculated using an online calculator (https://wnarifin.github.io/ssc/ssicc.html). A priori power calculation (ICC = 0.95, *p <* 0.05, power = 0.80) resulted in a sample size of 14 participants (Bonett 2002). Fourteen elite (i.e., professional and international‐level (McKay et al. 2022)) female water polo players (24.3 ± 3.2 years, playing experience > 5 years), who were previously familiarised with the WIST and selected in the Australian women’s national water polo team, provided written consent before participation. The athletes were familiarised with the experimental procedures and tested on two separate occasions separated by 48 h. WIST testing was completed at the same time of the morning (±1 h) to account for diurnal variation (Drust et al. 2005). Testing was completed during an Australian national team training camp in which moderate‐intensity training activities were performed between the two trials. Participants were asked not to perform strenuous exercise beyond what was required during the training camp. Athletes were asked to maintain the same food intake prior to both testing sessions. Ethical approval was granted by an institutional research ethics committee, approval number 5763.

### 2.3 Procedures

Before physical activity, participants completed an individualised 10‐min dry‐land warm‐up involving mobility and strengthening exercises prescribed by the team’s physical performance coach. Participants subsequently completed a general swimming warm‐up involving 200 m freestyle, 1 × 50 m breaststroke kick, 1 × 50 m eggbeater kick, 2 × 50 m freestyle separated by 45 s, 4 × 25 m with an open water turn at 12.5 m and 1 × 50 m at 90%–95% of maximum effort. After a 5‐min rest period immediately after the warm‐up, participants performed the WIST following previously established methods (Mujika et al. 2006). The test began with 4 shuttles at 1.03–1.36 m/s, followed by 7 shuttles at 1.43–1.46 m/s. Speed was subsequently increased by 0.05 m/s every 8 shuttles until a player could no longer maintain the required speed. Testing sessions were video recorded (Sony HDR‐CX405 HD Camcorder) to confirm that participants were provided appropriate warnings and removed from the test when applicable. All swimming activities were completed in a 25‐m outdoor pool (22°–24° [±1.5°] C) with similar outdoor temperatures on testing days (25° [±1.0°] C). Players were instructed and encouraged to provide a maximal effort on each occasion by the primary investigator.

Participants were equipped with the same individual heart rate (HR) monitor (Polar Verity Sense, optical HR sensor) for both trials, and HR_peak_ was recorded upon test completion. Participants were also asked to rate their perceived exertion immediately after the completion of testing based on the CR10 scale (Bourg et al. 1987). Standard instructions and anchoring procedures were explained before the initial testing session. A rating of 0 was associated with no effort (i.e., rest), and a rating of 10 was associated with a maximal effort.

Before each testing session, participants completed the Short Recovery and Stress Scale (SRSS). The SRSS is an abridged version of the Acute Recovery and Stress Scale that quantifies the recovery‐stress state of individuals on a physical, mental, emotional and overall level (Kellmann and Kölling 2019; Nassi et al. 2017). The objective of the SRSS was to determine whether participants were in the same physiological, mental, emotional and overall state between testing sessions. Participants were asked how they felt in the present moment for each category of recovery and stress (Kellmann and Kölling 2019). Each item of the questionnaire was considered a separate variable, and all 8 items were completed using a 7‐point scale: 0 (does not apply) to 6 (fully applies) (Kellmann and Kölling 2019).

### 2.4 Statistical Analyses

The homogeneity of variance for all data across playing positions was verified using Levene’s test (*F* [2, 14] = 2.52, *p* = 0.116). Normality of data distribution was assessed using the Shapiro–Wilk test. Test‐retest reliability of all normally distributed data was analysed using confidence intervals (CI). The intraclass correlation (ICC) was calculated using a two‐way mixed effects model with single measures for absolute agreement and interpreted as follows: > 0.90 = excellent, 0.75–0.90 = good, 0.50–0.74 = moderate and < 0.50 = poor (Koo and Li 2016). The coefficient of variation (CV) is presented as a percent of participants’ mean scores (Hopkins 2000a), calculated from the mean differences and SD of trials (Turner et al. 2015) and interpreted as follows: < 5% = good, 5%–10% = moderate and > 10% = poor (Duthie et al. 2003). The smallest worthwhile change (SWC), proposed to be significant during the assessment of performance markers due to measurement noise during testing, was calculated by multiplying the between‐subject SD by the standardised effect size (ES) of 0.2 (small), 0.5–0.6 (moderate) and 1.2 (large) (Comyns et al. 2023; Turner et al. 2015). Typical error (TE) was calculated and presented with 90% CI to quantify within‐subject variation and the ‘noise’ associated with the WIST (Hopkins 2000b; Lexell and Downham 2005; Lindberg et al. 2022; Weir 2005). The reliability of the WIST was interpreted according to the following conditions: acceptable if the ICC ≥ 0.75 with a CV ≤ 10%, moderate when the ICC < 0.75 or CV > 10% and poor with an ICC < 0.75 and CV > 10% (Edwards et al. 2022). Standard error of measurement (SEM) was determined, using the formula SD_pooled_ x √1−ICC (Weir 2005), to concomitantly understand the precision of individual WIST scores and construct the minimal detectable change (MDC), one‐tailed calculated as 1.645 x SEM x √2, with 90% CI in WIST scores (Bucher et al. 2023; Ettema et al. 2021; Weir 2005). The usefulness and practicality of the WIST was determined by comparing the SWC with the TE, such that test evaluation was deemed marginal if the TE was higher than the SWC; the test was rated ‘OK’ if the TE and SWC were similar; and if the TE was less than the SWC, an evaluation of ‘good’ was given to the test (Pyne 2003).

Ordinal data were treated as nonparametric and analysed with paired‐samples Wilcoxon‐ranked tests to calculate effect sizes (ES), interpreted as follows: *r* = 0.1 (small), *r* = 0.3 (medium) and *r* = 0.5 (large) (Coolican 2009). Statistical significance was set at *p* < 0.05. Pairwise comparison effect sizes were calculated and presented using Hedges’ *g*. The ES thresholds for Hedges’ *g* were interpreted as follows: ≤ 0.2 (trivial), > 0.2 (small), > 0.6 (moderate), > 1.2 (large), > 2.0 (very large) and > 4.0 (extremely large) (Hopkins et al. 2009). Statistical tests were analysed in R Studio, using packages ‘*readxl’*, ‘*psych’*, *‘rstatix’*, ‘*ggstatsplot’* (Patil 2021), *‘dunn.test’*, *‘ggplot2’* and ‘*effectsize’* (R version 4.3.1).

### 2.5 Part 2 – Validity

#### 2.5.1 Subjects

Eighteen elite‐level (24.4 ± 3.5 years), seven highly trained (21.6 ± 3.2 years) and 34 trained (13–17 years) (McKay et al. 2022) female water polo players provided written consent before participation. The subjects were different from Part 1 of the study and were from a wider water polo population; hence, the classification system used to define the playing standards was in accordance with McKay et al. 2022. Players under 18 were required to have representatives from their schools or club programmes, and their parent/guardian provided written consent before participation. Normal practice and competitive activities occurred on the days before testing sessions, with the WIST completed at the start of training sessions. Ethical approval was granted by an institutional research ethics committee, approval number 5763.

### 2.6 Procedures

Before physical activity, participants completed an individualised 10‐min dry‐land warm‐up (identical to that described in Part 1). Participants subsequently completed a general swimming warm‐up involving 20 self‐paced shuttle swims, 2 shuttles with a head‐up swimming position and 5 x shuttles with progressively increasing speeds. Participants were then familiarised with the WIST by completing the initial three test levels. After a 5‐min rest period following the warm‐up and familiarisation, participants performed the WIST following previously described procedures (see Part 1) (Mujika et al. 2006). All swimming activities were completed in a 25‐m outdoor pool (23°–25° [±1.0°] C) with outdoor temperatures of 18°–19° ± 0.5°C.

### 2.7 Statistical Analyses

Descriptive data for WIST scores across playing positions were presented as median (interquartile range [IQR]) with 95% CI. Positional differences in WIST scores were compared using a Kruskal–Wallis test with effect sizes calculated using epsilon‐squared and interpreted as follows: < 0.01 (very small), ≥ 0.01 < 0.06 (small), ≥ 0.06 < 0.14 (medium) and ≥ 0.14 (large) (Field 2013).

Descriptive data for WIST scores across playing standards are presented as mean ± SD in metres. The assumption of homogeneity of variance was violated per Levene’s test (*F* [4, 54] = 14.471, *p* < 0.01). The Kruskal–Wallis test with Dunn’s test using Bonferroni correction for pairwise comparisons was used to compare playing standards. Effect sizes for pairwise comparisons for post‐testing were calculated using standardised mean difference (SMD) and presented as Hedges’ *g*. The ES thresholds for Hedges’ *g* were interpreted as follows: ≤ 0.2 (trivial), > 0.2 (small), > 0.6 (moderate), > 1.2 (large), > 2.0 (very large) and > 4.0 (extremely large) (Hopkins et al. 2009). Statistical tests were analysed in Jamovi (version 2.4.12.0), SPSS (version 20.0; SPSS Inc., Chicago, IL) and R Studio, using packages ‘*psych’*, *‘rstatix’*, *‘tidyverse’* and ‘*effectsize’* (R version 4.3.1).

## 3 Results

### 3.1 Part 1

**FIGURE 1:**
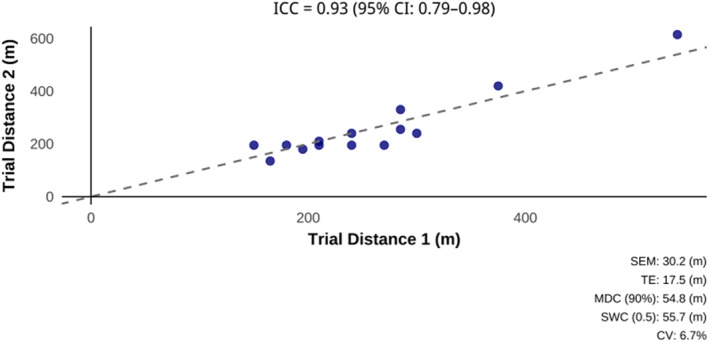
WIST test‐retest reliability measures using the distance completed. CI = confidence interval; CV (%) = coefficient of variation; ICC = intraclass correlation coefficient using a two‐way mixed effects model, absolute agreement (single measures); m = metres; MDC_90_ = minimal detectable change with 90% confidence intervals; SEM = standard error of measurement; SWC_0.5_ = smallest worthwhile change at 0.5 (moderate) effect size; TE = typical error.

Descriptive statistics for WIST scores for the elite‐level female water polo players are presented in Table 1. The test‐retest reliability measures, presented in Figure 1, for WIST scores displayed acceptable CV (6.7%) and good to excellent ICC (0.93 [0.79–0.98]), with a TE = 17.5 m [(11.6, 23.3)], SWC_0.2_ = 22.3 m, SWC_0.5_ = 55.7 m, MDC_90_ = 54.8 m and SEM = 30.2 m. Heart rate data showed good ICC (0.83 [0.61–0.83]) with no significant differences in HR_peak_ (*p* = 0.38) between testing sessions. In contrast, significant differences between SRSS scores (*z* = −3.11, *p* < 0.01, *r* = 0.89) were demonstrated between testing sessions.

**TABLE 1:** Descriptive statistics for WIST scores for the elite‐level female water polo players.

Measurements	Mean (*m*) (SD)	Mean difference (*m*) (95% CI)	*p*‐value	ES
WIST performance trial 1 (*n* = 14)	260.4 (100.8)	−3.2 (−28.7, 22.2)	0.79	0.07
WIST performance trial 2 (*n* = 14)	257.1 (124.9)			

*Note:* Values are presented as mean (SD); paired samples *t*‐test produced the *p*‐value; Hedges' *g* was used to report the effect size; statistical significance *p* < 0.05; CI = confidence interval; m = metres; SD = standard deviation.

### 3.2 Part 2

**FIGURE 2:**
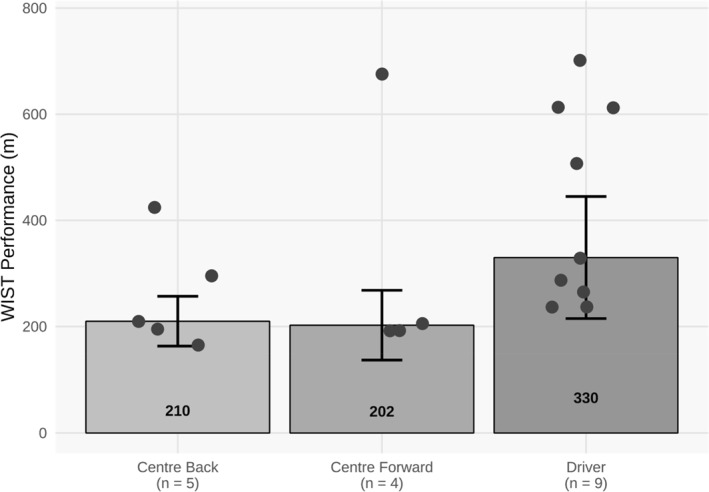
Positional differences in WIST performance in elite women's water polo players. Values are presented as median WIST performance. Positions included centre forward, centre back and driver. Nonparametric one‐way Kruskal–Wallis test produced the *p*‐value. *m* = metres; *n* = total number of participants in each playing position. (Statistical significance *p* < 0.05).

**FIGURE 3:**
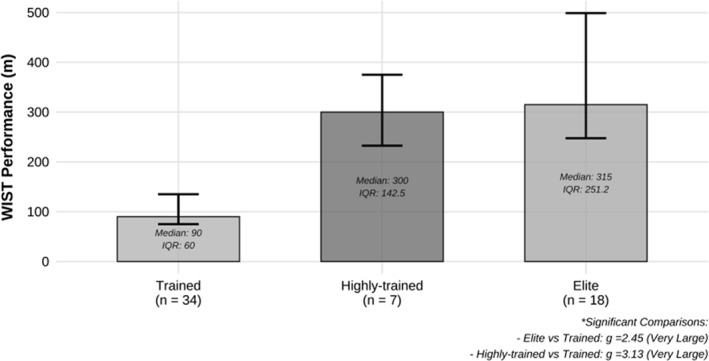
WIST performance comparisons across standards of competition. Kruskal–Wallis test with Dunn's test for pairwise comparisons. Elite standards include players currently in the national squad. Highly trained level included players currently representing their state team; trained‐level players included U14 to U17 teams. Effect size calculated using Hedges' *g*. Effect size interpretation was according to Hopkins 2000a and Hopkins 2000b. (Statistical significance *p* < 0.05).

The omnibus test showed no significant differences in WIST scores (*p* = 0.11, *ϵ* ^2^ = 0.14) among playing positions, with a medium effect size (Figure 2). The omnibus test showed that the WIST scores varied significantly across competitive levels, with the differences being large in magnitude (*p* < 0.001, *ϵ* ^2^ = 0.66). Significant differences were evident in WIST scores between elite‐level and trained (U14 to U17) players, with very large positive effect sizes (*p <* 0.01, *g* = 2.4). Significant differences in WIST scores were also demonstrated between highly trained and trained (U14 to U17) players with very large, positive effect sizes (*p <* 0.01, *g* = 3.1) (Figure 3).

## 4 Discussion

This study aimed to (a) establish the test‐retest reliability, standard error of measurement and minimum detectable change of the WIST in elite female water polo players, (b) investigate the validity of the WIST to determine positional differences within this population and (c) distinguish between competitive female playing standards. The main findings were that the WIST demonstrated acceptable test‐retest reliability. Additionally, the WIST was confirmed as a useful and valid test, capable of monitoring performance changes and identifying performance differences between competitive playing levels, respectively.

Findings showed that the WIST demonstrated acceptable test‐retest reliability (CV = 6.7%), equating to approximately one out‐and‐back shuttle (17 m). Additionally, the high ICC (0.93 [0.79–0.98]) indicates excellent consistency in performing the WIST across repeated measures. However, WIST scores demonstrated a higher degree of variability compared to previous research involving trained male water polo players (CV = 5.4%) (Mujika et al. 2006; Tan et al. 2009b). This is largely attributed to the study being conducted during a national training camp. The players were required to complete their normal training activities between tests, possibly resulting in lower scores of readiness and recovery. This is supported by the moderate, significant differences in recovery scores (*p* < 0.01, *r* = 0.89) reported between the first and second testing sessions. The decreased recovery scores may have contributed to the variability between the two tests. Nevertheless, the current findings suggest that the WIST is a reliable tool for assessing high‐intensity intermittent performance in elite female water polo players, even during intensified training periods.

In the current investigation, the SWC_0.2_ was 1.5 shuttles (22.3 m), and the SWC_0.5_ shuttles (55.7 m) and the MDC_90_ (54.8 m) were both approximately four shuttles. The usefulness of the WIST at detecting changes in performance was deemed to be good, given that the SWC_0.2_ (22.3 m) was greater than the TE (17.5 m). However, only the SWC_0.5_ was greater than both the SEM and MDC_90_; therefore, the WIST was only capable of detecting moderate changes in performance with both confidence and statistical significance. The higher noise and variations in WIST performance may be explained by the lower indicators of recovery and readiness scores across the initial days of the training camp. Importantly, practitioners conducting physiological performance tests should consider the measurement error for each test to interpret practically meaningful changes in performance (Lexell and Downham 2005; Lindberg et al. 2022; Weir 2005). As such, for sport scientists and performance support staff working in elite women's water polo wishing to quantify meaningful improvements in WIST performance, we suggest that changes > 55 m (∼4 shuttles) be considered as ‘real’. Because this is the first study to report the SWC and MDC during the WIST in elite female water polo players, making comparisons to similar findings in water polo becomes challenging. Nevertheless, given that the usefulness of the WIST is measured as good, practitioners working with elite women's water polo can develop performance standards for the WIST and monitor longitudinal changes in WIST performance after conditioning interventions.

The average heart rate during the WIST was 167 bpm across both trials. This was lower than HR responses previously reported in higher‐level Australian males (179 bpm) and females (180 bpm) performing the WIST (Mujika et al. 2006; Tan et al. 2009b). Tan et al. (Tan et al. 2009b) suggested that the short duration of the WIST may not allow sufficient time to significantly raise HR, as indicated by the lower values observed in this population of elite female players. It was also suggested that the turning ability of players may be a greater limiting factor in WIST performance than cardiorespiratory fatigue, representing a specific physical quality required during match play (Tan et al. 2009b). WIST scores were similar to those reported by Mujika et al. (Mujika et al. 2006). However, the HR_peak_ values were lower during and across trials (167 bpm vs. 177 bpm), which may either reflect heightened cardiorespiratory fitness levels among contemporary players compared to players competing nearly 20 years ago or the differences in the maximum heart rate and subsequent percentage HR_max_ attained of the individual players. Accordingly, Tan et al. (Tan et al. 2009b) reported similar HR_peak_ responses during the WIST in elite female players as those reported by Mujika et al. (Mujika et al. 2006), with concomitantly higher RPE scores after the test compared with the present group of female Australian national team members. Despite the differences in average HR, Tan et al. (Tan et al. 2009b) reported similar RPE scores (6.7 vs. 6.3). Tan et al. (Tan et al. 2009b) completed testing during the specific preparation phase, whereas this study assessed players during a training camp 2 months prior to an international competition. The differences likely reflect the increased physiological demands of modern‐day women's water polo, as current elite female players demonstrated a lower HR_peak_ for the same WIST performance score.

No significant differences in WIST performance were observed across playing positions, with keepers excluded from the sample. Although this study found a moderately strong association between WIST performance and playing position, with drivers achieving the best performance across positions, the overall nonsignificant findings are likely a result of the low number of participants in each positional group. In contrast, significant differences have previously been identified across positional groups, specifically between drivers and centre forwards (Mujika et al. 2006). Centre players (centre forward and centre back) are noted to spend twice as much time engaged in wrestling bouts and half the time performing sprinting efforts compared with perimeter players (Tan et al. 2009a). These differences may be attributed to the distinct positional demands and variations in body mass and swimming economy between the positions (Mujika et al. 2006; Platanou and Geladas 2006). As such, practitioners should consider differences in positional demands when using the WIST to assess high‐intensity intermittent performance in higher‐standard female players.

WIST performance was different between playing standards, with scores varying hierarchically across competition levels. Previous studies have similarly shown superior testing outcomes among players competing at higher levels compared to those at lower levels (Mujika et al. 2006; Tan et al. 2009b). Tan et al. (Tan et al. 2009b) reported substantial differences in WIST outcomes between members of the Australian women's national water polo team and players from a National Water Polo League club. These findings are consistent with earlier research showing higher WIST scores in elite compared to state‐level female players (Mujika et al. 2006). Although the studies similarly found small variations in physiological responses during the WIST across standards of play (Mujika et al. 2006; Tan et al. 2009b), previous research suggests that variations in cardiometabolic performance outcomes (i.e., aerobic and anaerobic capacities) may explain the contrasting results. Accordingly, higher‐level players may be able to prolong their time to fatigue and perform the WIST for longer durations due to superior fitness levels, supported by the present findings. Additionally, differences in morphological characteristics, training history, swimming speed and economy (Melchiorri, Padua, et al. 2010), as well as familiarity with the WIST itself, may have contributed to variations in testing outcomes across competition levels. Overall, these findings highlight the sensitivity of the WIST in identifying differences between players of varying competition levels (Mujika et al. 2006; Tan et al. 2009b).

It should be noted that the experimental activities reported in this study occurred during a training camp or during the competitive season. As such, participants only completed two WIST trials, possibly increasing the biases of the results (Hopkins 2000a). Additionally, because of the condensed playing schedule and heightened workloads typically associated with these periods, testing sessions were completed 48 h apart, and minimal modifications were able to be made to training activities. Thus, residual fatigue elicited by testing or training activities could have negatively affected the results. Ideally, these tests would be conducted with similar preparation conditions, but this can be difficult when working with elite athletes who have numerous training commitments. Additionally, other than HR_peak_, no physiological measures (e.g., blood lactate) were collected during testing, which somewhat limits the evidence supporting the validity of using the WIST to assess the match fitness of water polo players. Swimming performance during official matches was not assessed, and additional rule changes have occurred recently (2022–2024); therefore, the validity of the WIST to underpin swimming performance during competitive matches warrants further research considering the updated rule changes. The small sample of positional data available may limit the generalisability of the results, particularly with the interesting outlier in the centre forward group. Moreover, concomitant match performance indicators were not collected, further limiting the ability to evaluate the relationship between WIST outcomes and actual match performance. Further examination is needed to quantify present‐day match activities, investigate physiological indices of match fitness and compare these measures with WIST outcomes in a larger sample of competitive female players.

## 5 Practical Applications

The results of this investigation suggest that the WIST is a reliable test for elite female water polo players. The WIST demonstrated a good ability to detect changes in performance with an SWC (22.3 m) greater than the TE 90% CI (17.5 m) [(11.6, 23.3)]. However, considering the variation in and precision of WIST scores, as evidenced by the TE (17.5 m) and SEM (30.2 m), respectively, practitioners are encouraged to account for measurement error using ‘benchmarks’ greater than the SWC_0.5_ of 55.7 m, or ∼4 shuttles, to provide statistical assurance and confidence at a group level. Differences among playing standards, elite‐level, highly trained and trained, not playing position, were reflected in WIST scores, indicating the sensitivity of the test. Although test results could be interpreted on both a between‐ and within‐player basis, the former comparison should be made within particular playing positions, given the different demands placed on these individuals during match play (Botonis et al. 2019; D’Auria and Gabbett 2008; Mujika et al. 2006; Platanou and Geladas 2006; Smith 1998; Tan et al. 2009a). The collective findings suggest that the WIST is a useful tool as part of a performance testing battery for elite female water polo players.

## Funding

The authors have nothing to report.

## Conflicts of Interest

The authors declare no conflicts of interest.
